# Transcriptomics and Mendelian randomization studies reveal the critical role of Stanniocalcin-2 in linking perfluorinated compound-exposure to colorectal cancer

**DOI:** 10.3389/fpubh.2026.1768633

**Published:** 2026-04-28

**Authors:** Bingxin Li, Qianqian Yang, Jianghui Wu, Jiahui Zheng, Na Zhao, Zhaoyu Gao, Yang Luo, Rui Zhang, Shunjiang Xu

**Affiliations:** 1 Central Laboratory, The First Hospital of Hebei Medical University, Shijiazhuang, China; 2 Department of Pathophysiology, Hebei Medical University, Shijiazhuang, China; 3 Clinical Pharmacy Department, The First Hospital of Hebei Medical University, Shijiazhuang, China; 4 Hebei Key Laboratory of Critical Disease Mechanism and Intervention, Shijiazhuang, China; 5 Department of Information and Computing Science, Jinan University – University of Birmingham Joint Institute at Jinan University, Guangzhou, China; 6 Hebei Key Laboratory of Brain Science and Psychiatric-Psychologic Disease, Shijiazhuang, China

**Keywords:** colorectal cancer, perfluorinated compounds, risk model, Stanniocalcin-2, transcriptomics

## Abstract

Colorectal carcinoma (CRC) represents a major gastrointestinal malignancy with substantial global incidence, marked by elevated morbidity and mortality rates. Despite advancements in treatment, there is still considerable variation in prognosis, particularly with high recurrence rates, emphasizing the need for more precise prognostic tools. The present study is aimed at identifying central prognostic biomarkers linked to perfluorinated compound (PFCs) exposure and construct a prognostic risk model for CRC based on transcriptomic data. We analyzed transcriptome data from 638 CRC samples and 51 normal control samples from The Cancer Genome Atlas (TCGA) database, then identified a total of 3,230 PFC-associated genes. Candidate genes related to CRC prognosis were found by analyzing differences in gene expression, doing functional enrichment, and building a protein–protein interaction (PPI) network. We built a LASSO model with PRAME, CDKN2A, and STC2 and checked it using Kaplan–Meier analysis and ROC curves over time, while STC2 was further identified as the key mediator linking PFC exposure to CRC risk based on Mendelian randomization and experimental validation. This model could put patients into high-risk or low-risk groups, which gives doctors a new tool to plan personalized treatments. We also checked the immune setting, tumor mutation load, and likely response to immunotherapy, which further proved the model’s usefulness in the clinic. A Mendelian randomization (MR) analysis in two steps showed that higher levels of genetic risk for Perfluorooctanoic acid (PFOA) and Perfluorooctanesulfonic acid (PFOS) exposure raised CRC risk. STC2 was found to partly explain the mediator from PFOA to CRC, making up about 8.6% of the overall effect. We ran sensitivity checks, and the results still held, which means these cause-and-effect findings are strong. Finally, *In vitro* analyses verified that PFOA upregulates STC2 transcription in CRC cells. Our results suggest new ways to assess CRC prognosis and give scientific backing for treating cancers linked to the environment, based on how PFCs work in the body.

## 1 Introduction

Colorectal cancer (CRC) is a major type of digestive system cancer. For patients whose cancer has spread, the five-year survival rate is less than 20% (1, 2). Studies show that finding colorectal cancer early and using targeted treatments can greatly improve patients’ chances of survival (3, 4). In current clinical practice, commonly used prognostic genes are widely applied, but their limited sensitivity and specificity often fall short of meeting the demands of personalized precision medicine. Meanwhile, the heterogeneity and dynamic evolution of tumors further complicate the development of prognostic gene markers (5, 6). Against this background, finding new biomarkers with better specificity and more accurate prognostic power has become a key research goal, offering the potential to enable more precise diagnosis and treatment.

Due to their potential for bioaccumulation and persistence, PFCs are increasingly recognized as pollutants of concern (7). Since being introduced in the 1950s, PFCs have been widely used in metal industries, electronics manufacturing, and various industrial fields. Their widespread use has resulted in massive environmental releases. It is estimated that nearly 80% of the released perfluorocarboxylic acids can be directly linked to contamination during PFC production processes (8–10). Due to their strong carbon-fluorine bonds, these compounds are difficult to break down, leading to long-term accumulation in ecosystems (11). More and more animal studies show that PFCs can move through the body and may cause cancer. In rodent studies, PFCs get into the body through the gut and often build up in the liver, kidneys, and blood (12). Also, strong evidence links PFCs to the growth of malignant tumors. For example, PFOA can cause tumors in the testes, liver, and pancreas, and also raises the chance of breast cancer (13). In human urogenital system cancers, PFCs are connected to higher bladder cancer risk. Models based on PFCs work well to predict how the disease may develop (14, 15).

To achieve precise diagnosis and treatment for CRC, we must first break through the limits of traditional prognostic genes (16). PFCs, as potential cancer-causing pollutants, could point to new ways to study CRC’s molecular processes and develop better prognostic markers (17, 18). This could help move forward research that looks at how environmental exposure and tumor variety are connected.

However, we still do not clearly understand how PFCs and CRC are connected (19). Studies show that being exposed to PFCs may lead to more CRC cases and worse outcomes for patients (20, 21). MR analysis can also help find substances that might protect against cancer or raise its risk (22). To test this idea, we used open transcriptomic data and a combined bioinformatics approach to study PFC-linked gene networks in CRC in a systematic way. Based on this, we then used MR analysis and RT-qPCR to confirm PFC-related genes tied to CRC and checked how well they could help predict the disease’s outcome. This study aimed to find key genes and pathways regulated by PFCs, see how they relate to changes in the immune environment, and create a novel risk model to predict CRC outcomes. By linking environmental exposure to the molecular and immune traits of CRC, this work offers fresh insights into how the environment can drive cancer and helps advance more personalized tools for predicting patient outcomes.

## 2 Materials and methods

### 2.1 Data sources and preprocessing

This study based on transcriptome sequencing data from The Cancer Genome Atlas (TCGA) (23) and their clinical characteristics of CRC patients: 638 tumor samples (614 with complete survival information) and 51 normal controls. Samples with missing key clinical variables, including survival time and survival status, were excluded from subsequent prognostic analyses, and a complete-case analysis strategy was applied without data imputation. Settled GSE17537 (24), the Gene Expression Omnibus (GEO) database was used to obtain 55 CRC tumor samples containing clinical annotations for external validation.

Genes associated with PFCs were obtained from the Comparative Toxicogenomics Database (CTD), specifically targeting five key PFCs: perfluorononanoic acid (PFNA), perfluorooctane sulfonate (PFOS), PFOA, perfluorobutane sulfonate (PFBS), and perfluorodecanoic acid (PFDA), which were selected based on their high frequency of curated gene interactions and reported associations with colorectal cancer and related biological processes in the CTD database, as well as their widespread environmental occurrence and toxicological relevance. Other PFC homologs were not included due to limited curated evidence or insufficient gene interaction data available in the CTD database at the time of analysis. The CTD database was accessed in April 2025, and all data were retrieved from the publicly available resource.^1^ Assemblies were made of 3,230 genes that are related to PFC. In order to perform downstream analyses, raw count data was normalized and used R software (version 4.3.1).

### 2.2 Functional enrichment analysis

The enrichment analysis of PFC-associated genes using the R package “clusterProfiler” (v4.6.0) focused on GO terms and KEGG pathways. Logical significance was attributed to paths and functional categories with adjusted *p* < 0.05 (25).

### 2.3 Differential gene expression analysis

Differentially expressed genes (DEGs) between colorectal tumor and matched normal tissues were identified using the DESeq2 package (v1.38.3). Genes with an absolute log2 fold change greater than 2 and an adjusted *p* value below 0.05 were considered differentially expressed. Visualization through volcano plots and heatmaps was employed to represent gene expression differences (26). Candidate genes potentially related to PFC-driven CRC pathogenesis were identified by intersecting DEGs with the PFC-related gene set.

### 2.4 Establishment of PPI networks and detection of key regulatory genes

Candidate gene interactions were analyzed by constructing a protein–protein interaction (PPI) network using STRING (v11.5) with a confidence score threshold of 0.4. Visualization of the network was carried out using Cytoscape (v3.9.1). The CytoHubba plugin with the EcCentricity algorithm was applied to identify 20 pivotal genes characterized by maximal network centrality, designated as hub genes (27).

### 2.5 Prognostic gene screening

Univariate Cox proportional hazards analysis for overall survival (OS) in TCGA CRC patients was conducted using the R package “survival” (v3.3–1) that examined the hub genes. Identified genes with a *p* value below 0.1 and that satisfy the proportional hazards assumption (as verified by the Schoenfeld residuals test with a P above 0.05), were subsequently included in analyses. Prior to using R (25), the “glmnet” package (v4.1–4) was utilized to perform LASSO regression,.

### 2.6 Risk model development and validation

Genome-wide weights were used to add the products of gene expression levels for each patient in a risk score model, which was constructed using coefficients associating to LASSO. Inpatients were categorized by their median risk score, which classified them as either high-and low-risk. This was analyzed by the log-rank test and by using Kaplan–Meier survival curves in addition to generalized survival differences between the two groups. The “survivalROC” R package (version 1.0.3) provided time-based ROC curves for 1-, 3-, and 5-year time periods to test the model’s predictive accuracy, and the AUC values associated with them were calculated (19). The extraversion (external GSE17537 dataset (25)) was also used to verify the constructed risk score formulation.

### 2.7 Multivariate prognostic analysis and nomogram establishment

The OS of CRC patients was tested using univariate and multivariate Cox regression analyses, which adjusted for clinical variables like tumor stage and age, gender, and tumor classification and TNM classification. Multivariate Cox regression analysis was performed to evaluate whether the risk score remained an independent prognostic factor after adjusting for these available clinical variables. As a consequence, variables with a *p* value below 0.05 in the multivariate analysis were classified as independent prognostic factors (25). They constructed a nomogram from the “rms” R package (version 6.3–0) that accounted for not only the risk score but also important clinical factors, and estimated the OS probabilities for 1-, 3-, and 5-years. Predictive accuracy of the nomogram was measured not only by the concordance index (C-index) but also by calibration plots.

### 2.8 Pathway enrichment analysis by gene set enrichment analysis

Low-and high-risk groups were enriched differently in pathways analyzed through the use of Gene Set Enrichment Analysis (GSEA) to determine their pathways. The gene clustering algorithm was based on log2 fold change, and the R package “clusterProfiler” (v4.6.0) was utilized to analyze them. MSigDB provided the Gene Bank sequences for Hallmark and KEGG, which were also utilized as reference genes. The significance of paths was attributed to their normalized enrichment score (NES) exceeding 1.5 and an adjusted *p* value falling below 0.05 (28).

### 2.9 Immune microenvironment analysis

For CRC sample-derived immunonetic cells, xCell was used to measure proportions of immune cells and Wilcoxon rank-sum tests were used to assess infiltration differences between risk groups (*p* < 0.05 was considered statistically significant). Spearman correlation analysis was performed to assess associations between prognostic gene expression, risk scores, and immune checkpoint genes (29).

### 2.10 Tumor mutation burden analysis

Tumor mutation burden (TMB), representing the number of somatic mutations per megabase in tumor samples, was calculated to compare mutational differences between high- and low-risk groups. For this purpose, they used the R package “maftools” (v2.14.0) which is used to analyze mutation data from patients of the TCGA CRC. Waterfall plots were generated to visualize mutation profiles of the top mutated genes. Data was analyzed using Kaplan–Meier analysis, with a *p* < 0.05 for the prognostic impact of TMB (30).

### 2.11 TIDE immunotherapy response prediction

The Tumor Immune Dysfunction and Exclusion (TIDE) algorithm was applied to estimate the potential response to immune checkpoint blockade therapy. TIDE scores were computed for CRC samples in both risk groups to predict immune evasion mechanisms and therapy responsiveness. Differences in predicted responders between groups were assessed by Fisher’s exact test, and survival analyses based on TIDE scores were conducted. Statistical significance was set at *p* < 0.05 (31).

### 2.12 Drug sensitivity analysis and drug prediction

Drug sensitivity information, including IC50 values for chemotherapy and targeted drugs, was sourced from the Genomics of Drug Sensitivity in Cancer (GDSC) database. IC50 predictions for TCGA CRC samples were performed with the “oncoPredict” R package (v 0.2.0). Drug sensitivity differences between risk groups were evaluated by Wilcoxon rank-sum tests, with adjusted *p* < 0.05 considered significant (32). Additionally, drug prediction targeting prognostic genes was performed using the DSigDB database, and drug-gene interaction networks were constructed and visualized with Cytoscape (v 3.9.1) (33).

### 2.13 miRNA-mRNA-TF regulatory network construction

To investigate upstream regulation of prognostic genes, miRNAs and transcription factors (TFs) targeting these genes were predicted via the miRNet database. The predicted miRNA-mRNA and TF-mRNA interactions were combined to build a comprehensive miRNA-mRNA-TF regulatory network. This network was visualized using Cytoscape (v3.9.1), enabling identification of key regulatory elements potentially contributing to CRC progression (22, 34).

### 2.14 Statistical analysis

Analysis of statistical data was carried out using R software (version 4.3.1). Correlative analyses were carried out through the use of Student’s t-test or Wilcoxon signed-rank test using distribution for comparisons of continuous data. If the variables were categorical, then chi-squared or Fisher’s exact tests were used for testing purposes. These were then compared using Kaplan–Meier curves and time–to–event tests to determine survival ». Utilitarian cox proportional hazards regression models were utilized for both univariate and multivariate analyses. Use of the rank correlation coefficient, as prescribed, was found in Spearman’s study. If any two-tailed *p* value was less than 0.05, then the test yielded a statistical significance, unless otherwise noted.

### 2.15 Mendelian randomization analysis

We used two-step Mendelian randomization (MR) to test for possible causality between PFC exposure and CRC, with key prognostic genes (*PRAME*, *CDKN2A*, and *STC2*) considered as potential mediators. The inverse-variance weighted (IVW) method was used as the primary MR approach, supplemented by MR-Egger, weighted median, simple median, and mode-based estimators (35).

Genetic instruments for PFOA and PFOS were selected from published GWAS (GCST90265804 and GCST90265803, respectively), using a significance threshold of *p* < 1 × 10^–5^ (36) and minor allele frequency (MAF) > 0.01. Instrumental variables (IVs) were pruned for linkage disequilibrium (LD) using *r^2^* < 0.1 (22) and a 1 Mb (37) window. SNPs with F-statistics > 10 (38) were retained to reduce weak instrument bias. CRC GWAS summary statistics were obtained from the FinnGen R9 database (ID: finngen_R9_C3_COLORECTAL_EXALLC), including 6,509 CRC cases and 287,137 controls, in which CRC cases were defined based on International Classification of Diseases (ICD-10) codes (C18–C20), ensuring standardized and reliable disease classification. The FinnGen dataset was selected due to its large sample size, high-quality genotype data, standardized phenotype definitions, and homogeneous European ancestry, which helps reduce potential bias from population stratification in MR analysis.

Expression quantitative trait loci (eQTL) data for *PRAME* and *CDKN2A* were obtained from the IEU OpenGWAS project (IDs: eqtl-a-ENSG00000147889 and eqtl-a-ENSG00000185686, respectively), which are primarily derived from large-scale whole blood datasets. And protein QTL (pQTL) data for *STC2* were retrieved from deCODE Health, based on plasma proteomic measurements. For *CDKN2A* and *STC2*, methylation QTL (mQTL) data from the GoDMC consortium were used to assess methylation-mediated effects.

Data harmonization, allele alignment, and MR analysis were conducted using the TwoSampleMR R package (v0.5.10). Pleiotropy and heterogeneity were assessed using the MR-Egger intercept and Cochran’s Q test. Horizontal pleiotropy was further evaluated using the MR-PRESSO global test. The robustness of the results was tested using a leave-one-out analysis. The Steiger directionality test was applied to confirm the correct causal direction between exposure and outcome.

A mediation MR framework was applied, in which the effect of PFC exposure on CRC was decomposed into direct and indirect (mediated through gene expression) components. Only genes with at least three independent instruments were included in the mediation analysis.

### 2.16 Cell culture

SW480 and HCT116 cell lines were maintained in Dulbecco’s Modified Eagle Medium (DMEM; Gibco, Cat# 11965092) supplemented with 15% fetal bovine serum (FBS; Gibco, Cat# 10099141) and 1% penicillin–streptomycin (Gibco, Cat# 15140122) under standard culture conditions (37 °C, 5% CO2, humidified atmosphere) (39). PFOA (Sigma-Aldrich, Cat# 335–67-1, USA) was prepared by dissolution in DMSO. Specifically, dissolution of 4.14 g of PFOA in 10 mL of DMSO yielded a 10 mM stock solution. For treatment, 2 μL of this stock was diluted into 2 mL of culture medium, yielding a final concentration of 10 μM PFOA, which was used for subsequent experiments, and cells were treated for 24 h prior to RNA extraction. This concentration is commonly applied in *in vitro* studies to investigate the cellular effects of PFOA and to ensure detectable biological responses within a controlled experimental setting (14, 40).

### 2.17 Quantitative real-time reverse-transcription PCR (RT-qPCR)

Unizol Total RNA Extraction Kit (Genesand, Cat# RE704, China) was used as per the kit’s protocols to extract total RNA from tumor cells. The Reverse Transcription Kit (Vazyme, Cat# R323, China) was used as per the kit’s protocols to perform reverse transcription. Gene expression was detected using Roche LightCycler® 480 Real-Time Quantitative PCR System (Roche, Switzerland), and the amplification was performed according to the manufacturer’s instructions for the ChamQ Universal SYBR qPCR Master Mix (Vazyme, Cat# Q711, China). The thermal cycling protocol comprised an initial denaturation (95 °C, 30 s) followed by 40 cycles, each comprising a denaturation step (95 °C, 10 s) and an annealing step (60 °C, 30 s). The RT-qPCR assay was performed in triplicate. Relative expression levels of target genes, normalized to GAPDH, were calculated using the 2^(-ΔΔCt) method. The ΔΔCt was defined as: ΔΔCt = (Ct_target - Ct_GAPDH)_experimental - (Ct_target - Ct_GAPDH)_control (41). The primer sequences for the amplification of PRAME, CDKN2A, STC2, and GAPDH are listed in Table 1.

**Table 1:** The sequences of the primers used in RT-PCR assay.

Gene	Primers	Sequences
STC2	Forward	CACTGTTTGGTCAACGCTGG
	Reverse	AGCGTGGGCCTTACATTTCA
CDKN2A	Forward	CACCGCTTCTGCCTTTTCAC
	Reverse	ATGAAGTCGACAGCTTCCGG
PRAME	Forward	CTGGAAGCTACCCACCTTGG
	Reverse	GGATGTGGGAGAGGAGGAGT
GAPDH	Forward	GGTGAAGGTCGGTGTGAACG
	Reverse	CTCGCTCCTGGAAGATGGTG

## 3 Results

### 3.1 Identification of differentially expressed genes and PPI network analysis

**Figure 1:**
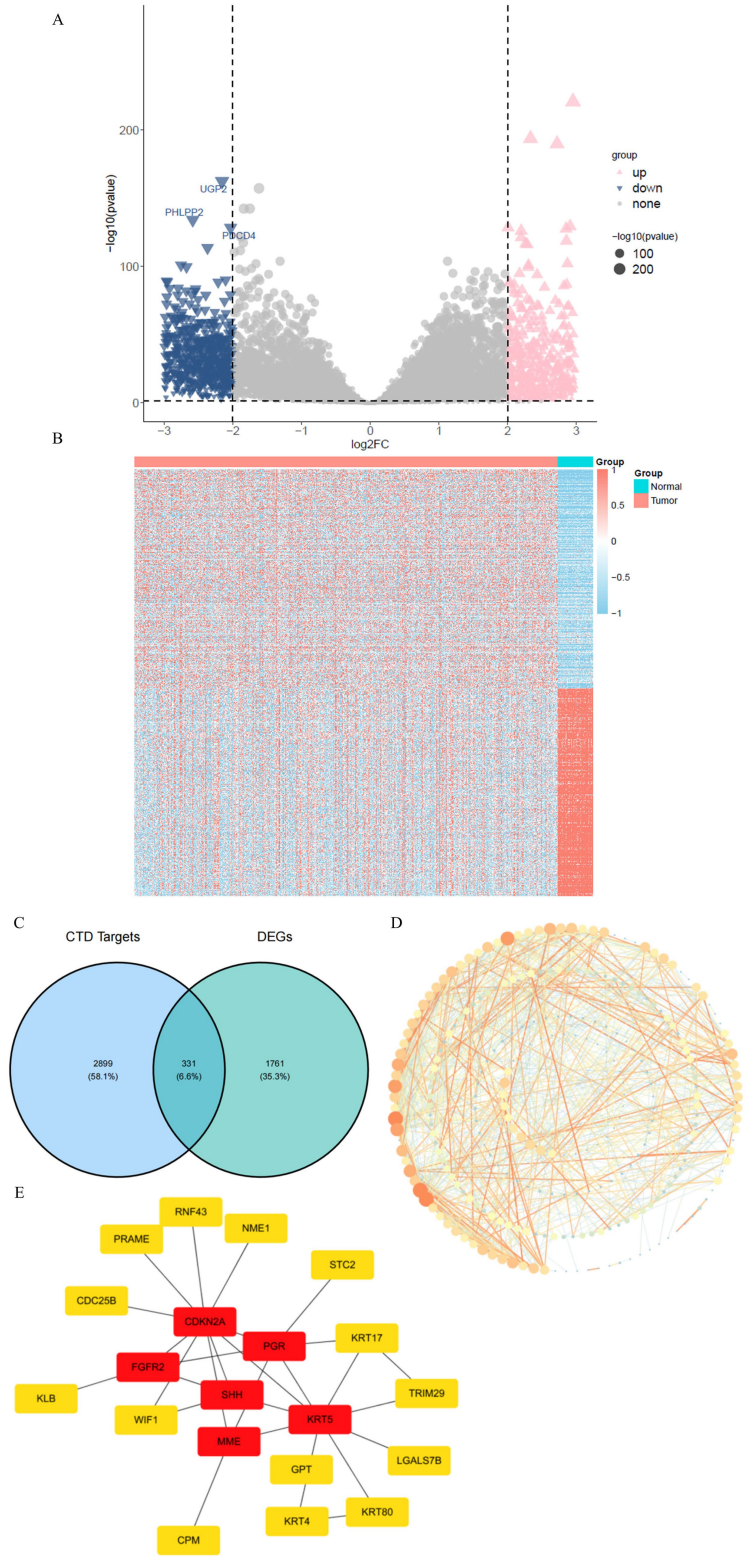
Identification of DEGs and PPI network analysis. **(A,B)** Volcano plot and heatmap depict distinct gene expression profiles between CRC tumors and normal tissues, with clear segregation of significantly upregulated and downregulated genes (|log2FC| > 2, adj. *p* < 0.05). Key genes labeled highlight potential biomarkers. **(C)** Venn diagram demonstrates overlap between DEGs and PFC-related genes, identifying 331 candidate genes potentially linking environmental exposure to tumor biology. **(D)** The STRING-derived protein–protein interaction network visualizes functional connectivity among candidate genes, revealing a densely connected module indicative of core molecular interactions. **(E)** Hub gene analysis via EcCentricity highlights 20 central nodes within the PPI network, including CDKN2A and KRT5, that may serve as pivotal regulators in CRC progression.

One thousand and seventy eight up-regulated genes and 1,014 down-regulated genes (2092 DEGs) were identified in CRC tumor tissues and adjacent normal tissues using the DESeq2 software package (Figures 1A,B). Combining 323 candidate genes from the CTD database with DEGs that could be involved in PFC-related CRCs resulted in the identification of 331 candidate genes (Figure 1C). By typing one or more candidate genes into the STRING database, one could create a protein interaction network with 265 nodes and 994 edges with a confidence interval of 0.4 (Figure 1D). Cytoscape software employed the CytoHubba plugin to conduct centrality analysis by deriving the top 20 central genes using the EcCentricity algorithm. These genes, including CDKN2A, KRT5, FGFR2, and SHH, were chosen for the analysis’s downstream prognostic function (Figure 1E).

### 3.2 Functional enrichment of PFC-related genes

**Figure 2:**
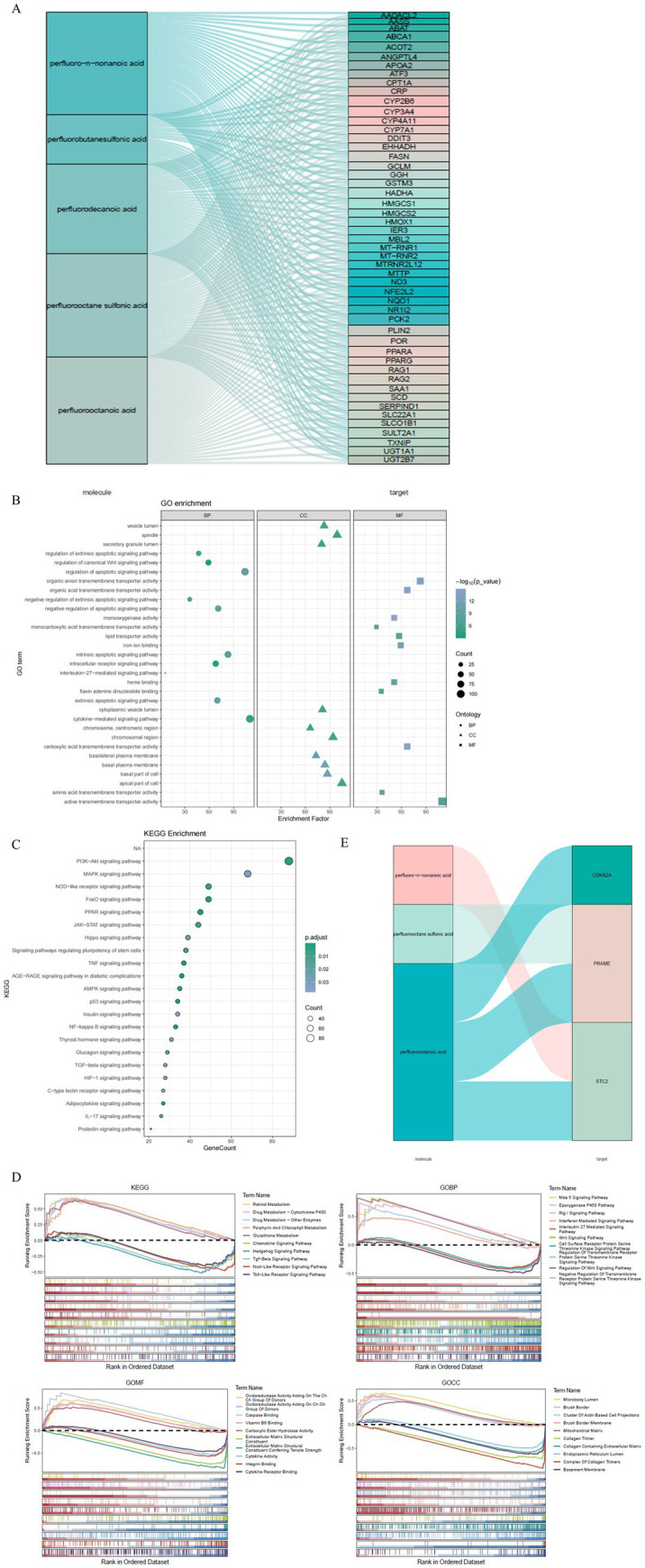
Functional enrichment and PFC–gene interaction analysis. **(A)** A Sankey diagram reveals the complex associations between five representative perfluorinated compounds and their corresponding target genes, highlighting the top 20 genes with the highest interaction frequencies. This network underscores key molecular targets potentially mediating PFC effects in CRC. **(B)** GO enrichment analysis categorizes PFC-related genes into biological processes, cellular components, and molecular functions, emphasizing pathways such as apoptotic regulation and canonical Wnt signaling critical to tumor development. Dot size corresponds to gene count; color intensity reflects enrichment significance. **(C)** KEGG pathway enrichment identifies multiple cancer-relevant and immune regulatory pathways including PI3K-Akt and JAK–STAT signaling, which may underlie PFC-associated colorectal carcinogenesis. Bubble size represents gene numbers involved; color gradient denotes adjusted *p*-values. **(D)** GSEA enrichment plots for selected KEGG and GO terms demonstrating differential pathway activation between high- and low-risk groups. Key pathways include metabolic processes, immune signaling, and receptor-mediated activities relevant to CRC pathophysiology. The running enrichment score and gene rank distributions illustrate the contribution of leading-edge genes. **(E)** Sankey diagram depicting the interaction between three prognostic genes (PRAME, CDKN2A, STC2) and their linked perfluorinated compounds, suggesting potential molecular mechanisms linking PFC exposure to CRC prognosis.

In CRC to investigate the linkage between PFC components and target genes in. In this case, a Sankey diagram illustrates the connections between five PFC compounds and genes that are related. Twenty highly interacting genes are depicted (Figure 2A). From this analysis of 3,325 core terms, GO enrichment analysis yielded findings. PFC-associated genes significantly enriched in biological processes: Wnt signaling, apoptotic signaling, cytokine-mediated signaling, and intracellular receptor signaling (Figure 2B). KEGG pathway analysis revealed 134 pathways that were significantly enriched, including JAK–STAT, PI3K-Akt, NF-B, FoxO, and TNF signaling pathways, among others, and classified cellular component and molecular function categories (Figure 2C). Hence all pathways are associated with both CRC development and immune regulation. Determinants of CRC pathogenesis and progression were found to be linked to genes related to PFC, which play a role in various important signaling pathways.

### 3.3 GSEA pathway enrichment in risk groups

Investigate and utilize GSEA pathway enrichment analysis to identify pathways in TCGA-CRC samples through gene expression domains (BP, CC, MF, and KEGG). Pathways that are significantly enriched in both the high and low-risk groups are the focus. Results revealed highly enriched pathways including glutathione metabolism, chemokine signaling, Hedgehog signaling, TGF-*β* signaling, Nod-like receptor signaling, Toll-like receptor signaling, RIG-I-like receptor signaling, interferon-mediated signaling, interleukin-27-mediated signaling, and Wnt signaling. (Figure 2D). Relative perfluorinated compounds were found to be associated with the prognostic gene-PFC interaction network involving three prognostic genes. It can be inferred that there is a mechanistic association between CRC prognosis and environmental exposure (Figure 2E).

### 3.4 Prognostic gene screening and risk model construction

**Figure 3:**
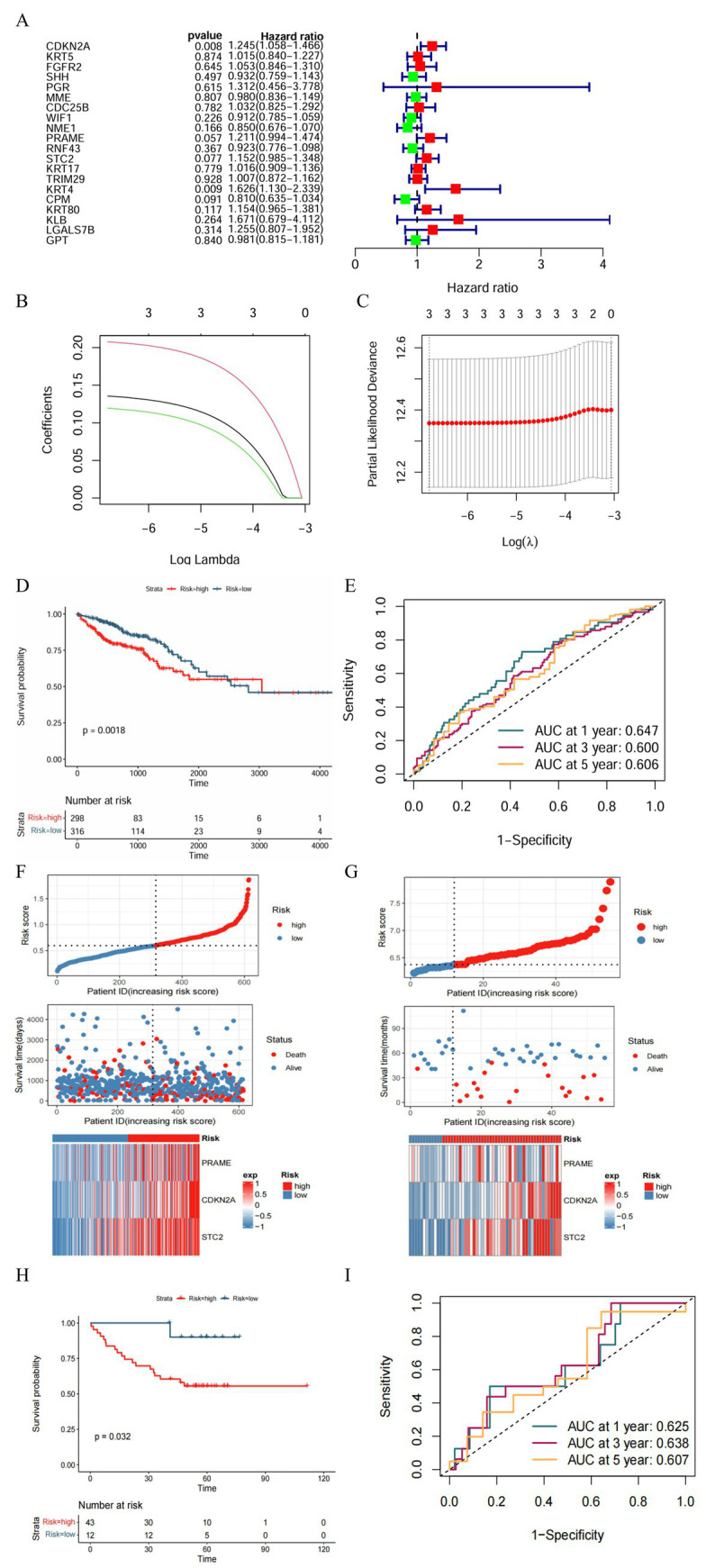
Prognostic gene screening and risk model construction. **(A)** Forest plot of univariate Cox regression identifies hub genes significantly associated with overall survival in TCGA CRC patients, guiding subsequent gene selection. **(B,C)** LASSO regression determines the optimal prognostic gene set by penalizing coefficients, balancing model complexity and predictive performance. **(D)** Kaplan–Meier survival curves illustrate markedly poorer survival in the high-risk group compared to low-risk patients, validating the prognostic value of the gene signature. **(E)** Time-dependent ROC curves demonstrate the model’s moderate to strong accuracy at 1-, 3-, and 5-year survival predictions. **(F)** Risk score distribution shows a clear separation between low- and high-risk groups, correlating with patient outcomes. **(G)** Survival status plot correlates risk scores with mortality events, illustrating higher death rates in high-risk patients. **(H,I)** Independent validation in the GSE17537 cohort confirms the robustness of the risk model with consistent survival disparities and predictive performance.

A systematic test utilizing univariate Cox proportional hazards regression to determine the prognostic value of 20 central genes and evaluate their correlation with overall survival in TCGA colorectal cancer. Respectively-adjusted proportional hazards assumptions led to the choice of selecting genes with *p* < 0.1; LASSO regression tested for and determined three prognostic genes: PRAME, CDKN2A, and STC2(Figures 3A–C). Using LASSO coefficients weighted gene expressions, a risk score model was developed: Risk score = 0.1356 × *PRAME* + 0.2078 × *CDKN2A* + 0.1190 × *STC2*.

Based on their cutoff score of 0.596, the TCGA cohort of patients was divided into two groups: those with low-risk and high-risk outcomes. The statistical analysis revealed that the low-risk group had better chances of survival than the high-risk group, as shown by Kaplan–Meier survival analysis (*p* < 0.01) (Figure 3D). Those who scored higher were more likely to die when mortality rates were assessed using the scoring distribution and survival status plots (Figure 3F). Determining efficacy: Time-sensitive ROC curves indicated that the model’s predictive efficacy was confirmed, with AUC values for survival predictions over 1 year, 3 years, and 5 years all exceeding 0.6 (Figure 3E).

The depletion in survival probability for high-risk patients in the GSE17537 gene expression database cohort was replicated through independent validation using the same risk formula and an optimal cutoff at 6.373(Figure 3H). The mortality rate was positively linked to the risk score used in the validation cohort, which was aligned with the survival status analysis (Figure 3G). Thus the AUC predicted for survival over the first year (Figure) is 0.625 while the survival predictor has the AUC for the next year (ROC analysis) for 1-year, 3-year, and 5-year spans, demonstrating the stability and universality of this prognostic feature (Figure 3I).

### 3.5 Independent prognostic value and nomogram performance

**Figure 4:**
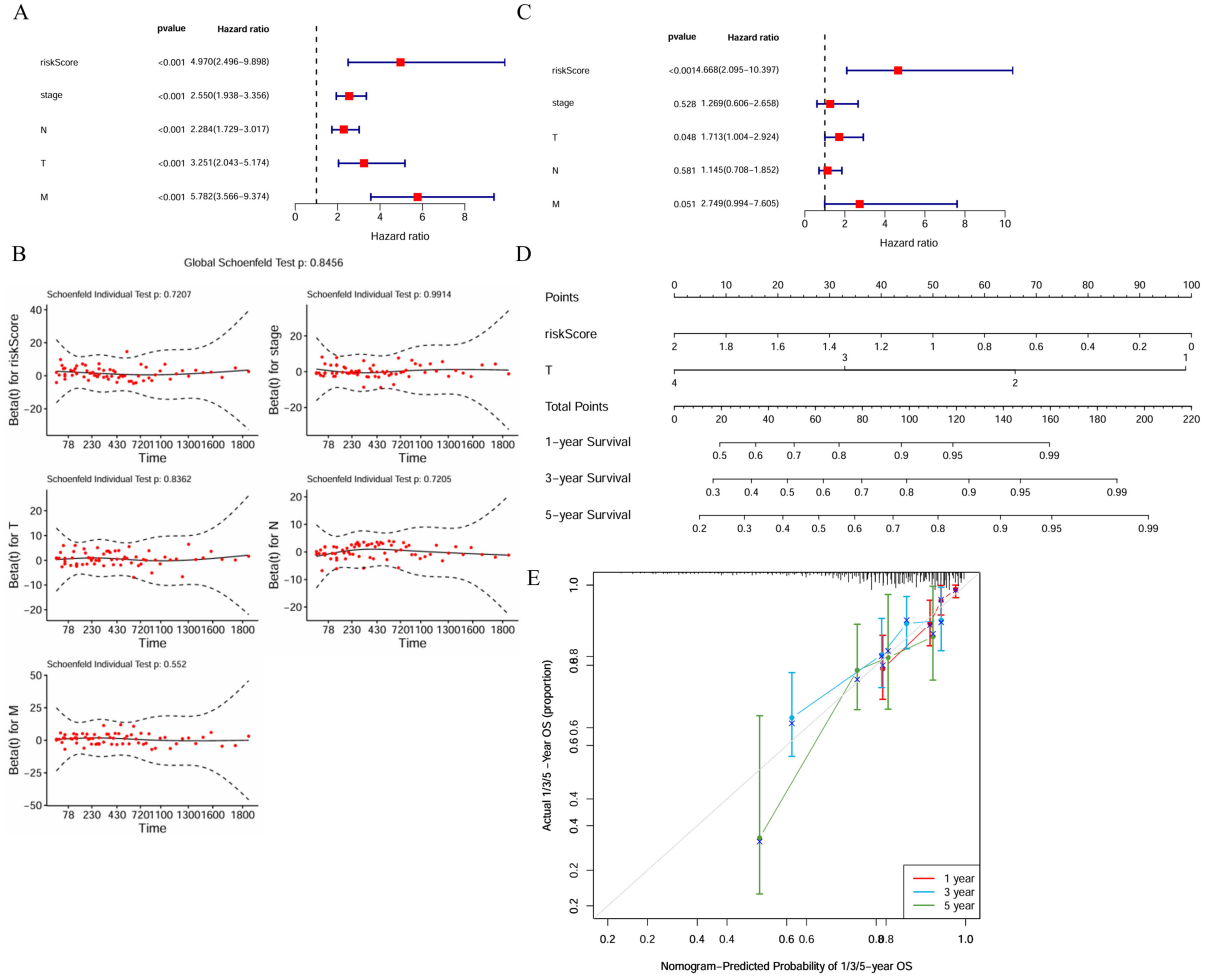
Independent prognostic value and nomogram performance. **(A)** Univariate Cox regression analysis identifying clinical variables and risk score significantly associated with OS in TCGA CRC patients. Risk score exhibits the strongest hazard ratio, indicating robust prognostic relevance. **(B)** Schoenfeld residual tests validating the proportional hazards assumption for risk score and clinical covariates, confirming model reliability. **(C)** Multivariate Cox regression analysis confirming risk score and tumor T stage as independent prognostic factors influencing patient outcomes. **(D)** Nomogram integrating risk score, age, and T stage to predict 1-, 3-, and 5-year OS probabilities, enabling individualized survival estimation. **(E)** Calibration curves showing close alignment between predicted survival probabilities from the nomogram and actual patient outcomes at various time points, highlighting model accuracy.

The development of a composite nomogram that incorporates risk scores, age, and tumor T-stage was intended to improve clinical utility by estimating the odds of survival lasting 1, 3, and 5 years (Figure 4D). Through testing, this model’s C-index was determined to be 0.745. At each observation time point, there is a well-defined correlation between predicted and actual survival outcomes, as evidenced by calibration curves that exhibit remarkable predictive performance (Figure 4E). The distribution of risk scores (Figure 4A), survival status of patients (Figure 4B), and the expression heatmap of prognostic genes (Figure 4C) illustrate the stratification performance of the constructed model.

### 3.6 Immune microenvironment differences

**Figure 5:**
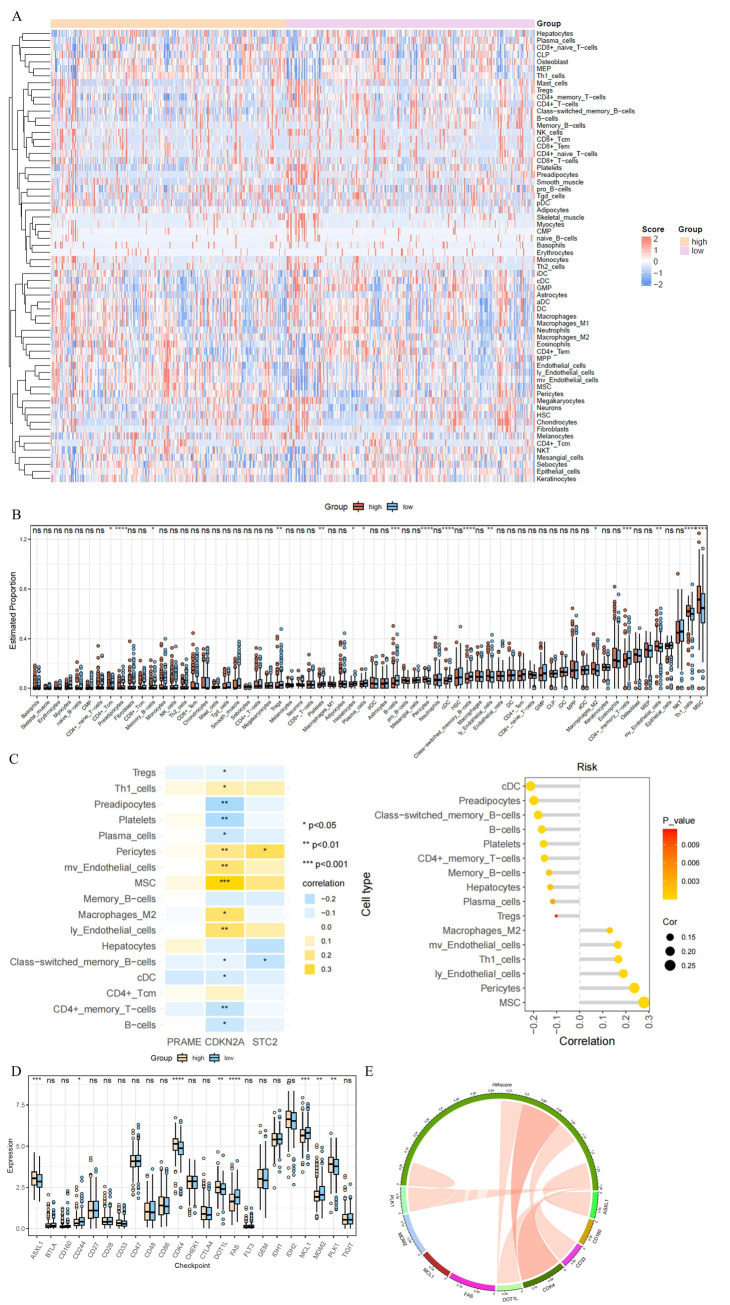
Immune microenvironment differences between risk groups. **(A)** Heatmap visualizing the differential abundance of diverse immune cell types between high- and low-risk groups based on xCell estimates, with hierarchical clustering illustrating distinct immune infiltration patterns. **(B)** Boxplots quantifying immune cell proportions with statistical comparisons, highlighting cell types significantly enriched or depleted in risk groups (e.g., M2 macrophages, Th1 cells). **(C)** Correlation heatmap illustrating associations between prognostic gene expression and immune cell infiltration scores, suggesting immune regulatory roles. **(D)** Boxplots showing differential expression of immune checkpoint genes across risk groups, with several checkpoints (e.g., FAS, MDM2) exhibiting significant upregulation in high-risk patients. **(E)** Circular diagram illustrating correlations between risk score and immune checkpoint gene expression, highlighting potential immune evasion mechanisms in high-risk CRC.

Significant differences between high-risk and low-risk groups were identified in 17 immune cell populations obtained from TCGA colorectal cancer samples analyzed using the xCell algorithm. CD4 + central memory T cells, M2 macrophages, and memory B cells showed marked disparities, while plasma cells and regulatory T cells (Tregs) exhibited intergroup variations. Different states of differentiation were observed in conventional dendritic cells, as well as in B cells and CD4 + memory T cells. Enrichment levels were significantly higher among both high-risk and M2 macrophage groups among the populations of Th1 cells, indicating significant distribution. Among the low-risk group, plasma cell infiltration showed an increase, which was a reflection of the risk-stratified immune landscape changes (Figures 5A,B).

Increased Th1 cell infiltration and myoendothelial cell responses are synchronized with positive expression of CDKN2A in M2 macrophages. Conventional dendritic cells and CD4 + memory T cells exhibit negative correlations, while B cell infiltration shows opposite associations. Within the lymphatic endothelial cell regulatory network (Figure 5C).

Thyroid function is influenced by immune checkpoints, and the expression of specific checkpoint molecules is linked to risk scores. Actually, immune suppressive mechanisms such as immune suppression involve both FAS and PLK1, with MDL1 and MDM2 showing differential expression (Figure 5E). Experts cautioned that FAS and MDM2 expression intensity differed markedly among patients, with significant expression gaps(*p* < 0.05) across eight checkpoints, including MCL1 and CD160, indicating immune suppression mechanisms in these diseases (Figure 5D). Patients’ prognosis trajectories are profoundly influenced by the modified immunological framework, which includes differentiated immune checkpoint expression patterns, and ultimately drives tumor immune escape.

### 3.7 TMB analysis and prognostic relevance

**Figure 6:**
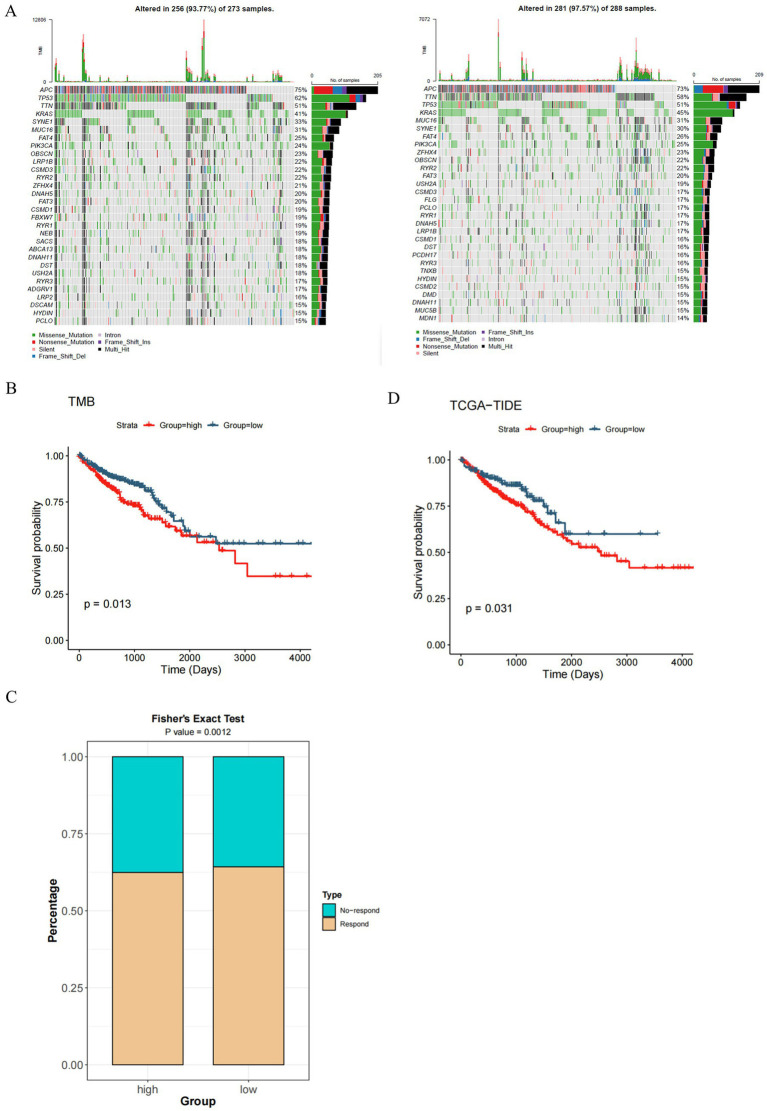
Immunogenomic and immunotherapy landscape of CRC risk groups **(A)** Waterfall plots depicting mutation profiles of the 30 most frequently mutated genes in high- and low-risk groups. Notable driver genes such as APC, TP53, and KRAS show distinct mutation patterns and frequencies. **(B)** Kaplan–Meier survival curves demonstrating significantly reduced overall survival in patients with high TMB, underscoring the prognostic value of mutation burden in CRC. **(C)** Comparison of response rates to immunotherapy between high- and low-risk groups based on TIDE classification. The proportion of responders is significantly higher in the low-risk group (*p* = 0.0012, Fisher’s exact test), suggesting better immunotherapy efficacy in this subgroup. **(D)** Kaplan–Meier survival curves demonstrate that patients with lower TIDE scores have significantly improved overall survival compared to those with higher scores (*p* = 0.031), highlighting TIDE’s utility in stratifying CRC patients for immunotherapy outcomes.

To compare differences in mutation between high-and low-risk patient cohorts, TCGA CRC samples were used to quantify TMB. High risk groupings showed significantly higher mean mean differences (*p* < 0.05) (Figure 6A). Unusual alterations in key oncogenes were observed through mutational analysis in both cohorts, with APC, TP53, TTN, and KRAS being mutated frequently. Outcomes were lower in patients with high TMB as in the survival analysis (*p* < 0.05) (Figure 6B). The evidence points to the fact that high TMB acts as a “negative prognostic biomarker” for CRC and enhances stratification ability when used with risk models.

### 3.8 TIDE analysis of immunotherapy response

The TIDE algorithm revealed significant differences in TIDE scores between high- and low-risk groups (*p* < 0.05) (Figure 6C). Patients in the high-risk group exhibited higher TIDE scores—suggesting increased probability of immune evasion—corresponding to reduced responsiveness to immune checkpoint inhibitors. Survival analysis established TIDE’s clinical value: higher TIDE scores correlated with decreased overall survival (*p* < 0.05) (Figure 6D). These findings highlight the utility of integrating TIDE into prognostic risk models to enhance the prediction of immunotherapy efficacy in CRC patients.

### 3.9 Drug sensitivity and prediction

**Figure 7:**
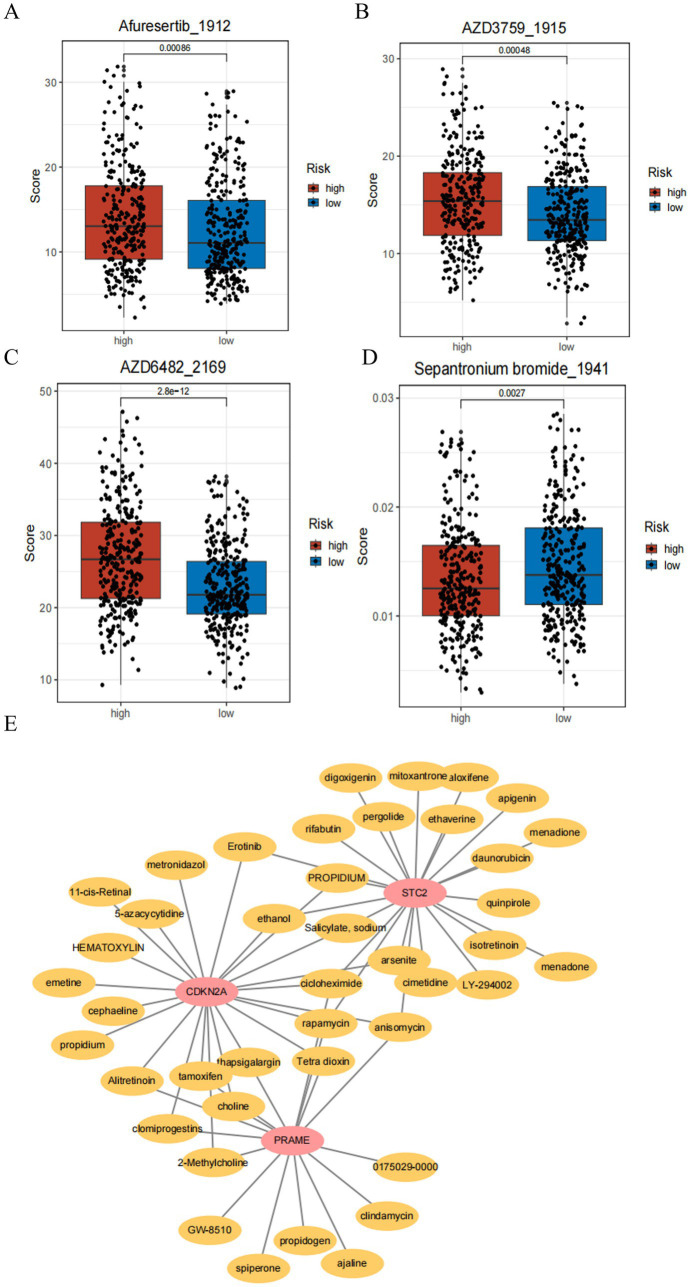
Drug sensitivity analysis and drug–gene interaction network. **(A–D)** Boxplots depict significantly lower predicted IC50 values for drugs Afuresertib, AZD3759, AZD6482, and Sepantronium bromide in the low-risk group compared to high-risk patients, indicating greater sensitivity to these agents. Statistical significance is indicated by adjusted *p*-values. **(E)** Network visualization showing predicted interactions between three prognostic genes (PRAME, CDKN2A, and STC2) and candidate drugs. In the network, pink nodes represent drugs and red nodes represent mRNAs. This network highlights extensive drug-gene relationships, suggesting potential targeted therapies customized to gene expression profiles.

The drugs were assessed through drug sensitivity testing using TCGA-CRC samples and the data on 198 drugs (and their normalized expression levels) is stored in the GDSC database. The calculations for chemotherapy drugs and targeted therapies were made possible by using the oncoCpredict software, which measures IC50 values for each patient. Comparisons were made using the Wilcoxon test, which compared IC50 levels of groups with high and low risk. Adjusted *p*-values for 19 drugs were significantly lower than 0.05—SB216763, IAP-5620, doramapimod, KU-55933, AZD6482; Lapatinib, RO-3306, AZD3759, and erlotinib also showed significant differences (Figures 7A–D).

Future therapeutic strategies for prognostic genes were studied by using association databases to predict drugs. A complex interaction network, consisting of 3 prognostic genes and 41 potential drugs, was identified, with 110 nodes exhibiting 77 interactions. The interaction graph below shows the specific interactions: PRAME was involved in 17 drugs, CDKN2A was involved in 23 drugs, and STC2 had the most extensive drug interaction, 37 drugs (Figure 7E).

### 3.10 miRNA-mRNA-TF regulatory network

**Figure 8:**
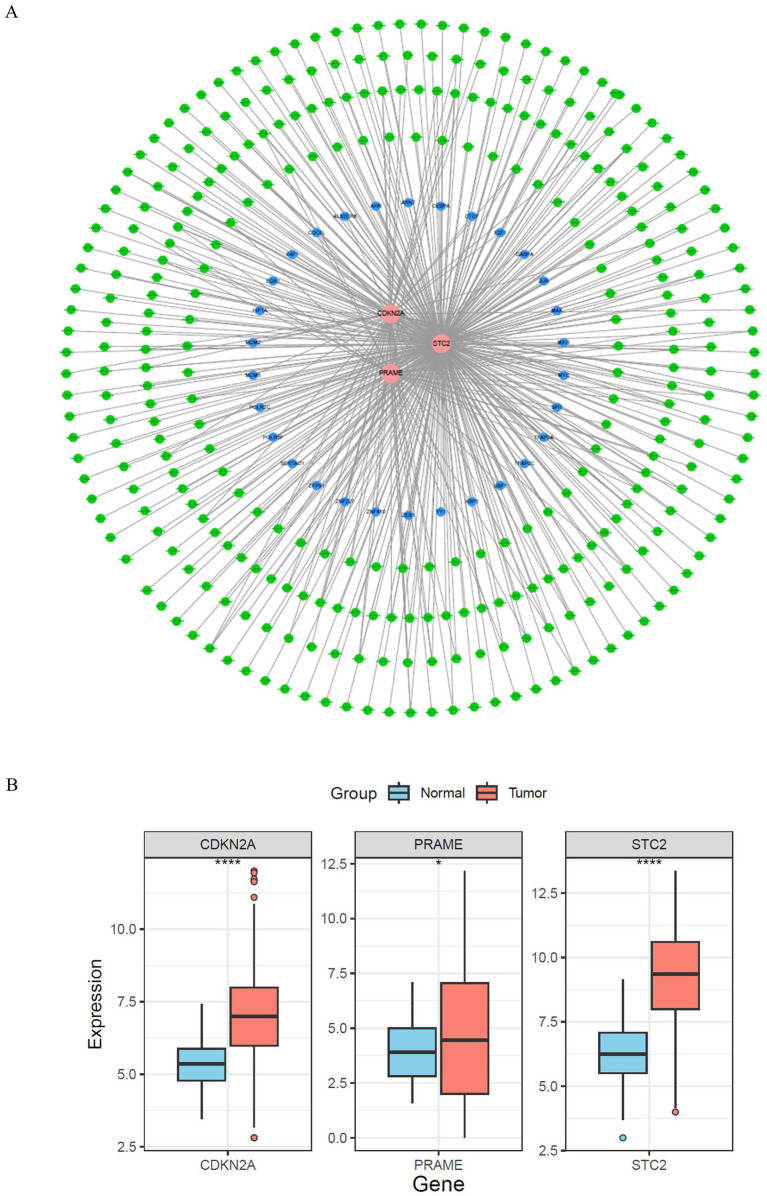
Regulatory network and expression validation of prognostic genes. **(A)** Visualization of the comprehensive regulatory network integrating three prognostic genes (red nodes), 381 miRNAs (green nodes), and 36 transcription factors (blue nodes). The dense web of interactions reflects the complexity of upstream regulatory mechanisms potentially driving CRC pathogenesis and influencing prognosis. **(B)** Boxplots illustrating significantly elevated expression levels of CDKN2A, PRAME, and STC2 in tumor tissues relative to adjacent normal tissues. Statistical significance at the level of *p < 0.05, **p < 0.01, ***p < 0.001, ****p < 0.0001), confirming the upregulation of these genes in colorectal cancer and supporting their roles as prognostic biomarkers.

A visualization of this regulatory network was constructed, comprising 354 nodes and 417 interaction edges: 3 prognostic genes, 381 miRNAs, and 36 TFs constitute these nodes. While MYC was ranked as the TF with the highest degree of connectivity according to network topology analysis. MRNAs that were more closely linked to each other, such as hsa-let-7a-5p and hsa-let-7b-5p, were significantly more connected than STC2, which was the most connected mRNA. However, mutually regulatory associations were observed between MYC, hsa-let-7a-5p, hsa-let-7b-5p, and STC2 (Figure 8A). The miRNA-mRNA-TF network could be a crucial factor in the pathogenesis of CRC.

### 3.11 Expression validation of prognostic genes

Three prognostic genes were identified in TCGA colorectal cancer samples, namely PRAME, CDKN2A, and STC2. Surrounding normal tissues did not exhibit a statistically significant increase in gene expression, with tumor tissues being more active (*p* < 0.001) (Figure 8B). The substantiation of the heightened expression of prognostic genes in colorectal cancer supports their crucial role in tumor growth and patient outcome.

### 3.12 STC2 mediates PFOA-induced CRC risk: evidence from two-step Mendelian randomization

**Figure 9:**
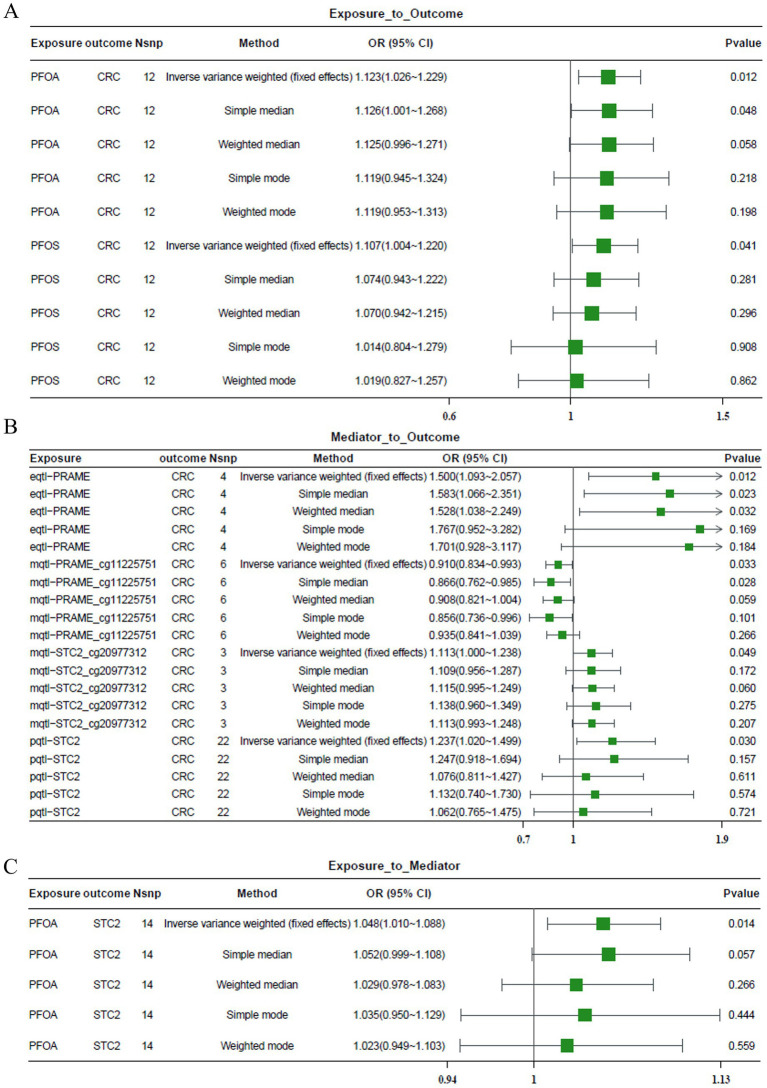
Two-step MR analysis of PFC–CRC mediated by PGs. **(A)** MR estimates of the causal effect of PFOA and PFOS exposure on CRC risk. Both exposures showed increased CRC risk (OR > 1) using the IVW method. **(B)** Causal effects of prognostic genes (PRAME, CDKN2A, STC2) on CRC. STC2 exhibited a significant positive association with CRC risk. **(C)** MR analysis showing PFOA exposure increases STC2 protein levels (pQTL), supporting a mediation role for STC2 in the PFOA–CRC pathway. OR and 95% confidence intervals (CI) were derived using the IVW method. OR > 1 indicates increased risk. SNP count, method, and *p*-values are provided for each comparison.

Pursuing a two-step MR analysis with PRAME, CDKN2A, and STC2 as mediators was utilized to investigate the causality of PFCs in CRC (Figures 9A–C).

The first-stage MR analysis revealed positive associations between PFOA and PFOS exposure and colorectal cancer risk. IVW estimates showed an odds ratio (OR) greater than 1 for both PFOA and PFOS exposures, indicating possible adverse effects (Figure 9A). The analysis concluded that STC2 (measured through pQTL) was the sole causal link between CRC and STC (IVW OR > 1; Figure 9B). The resulting third step showed a positive causal relationship (IVW OR > 1; Figure 9C) between PFOA and expression of STC2. These findings suggest that STC2 may act as a key mediator linking PFOA exposure to CRC risk.

**Figure 10:**
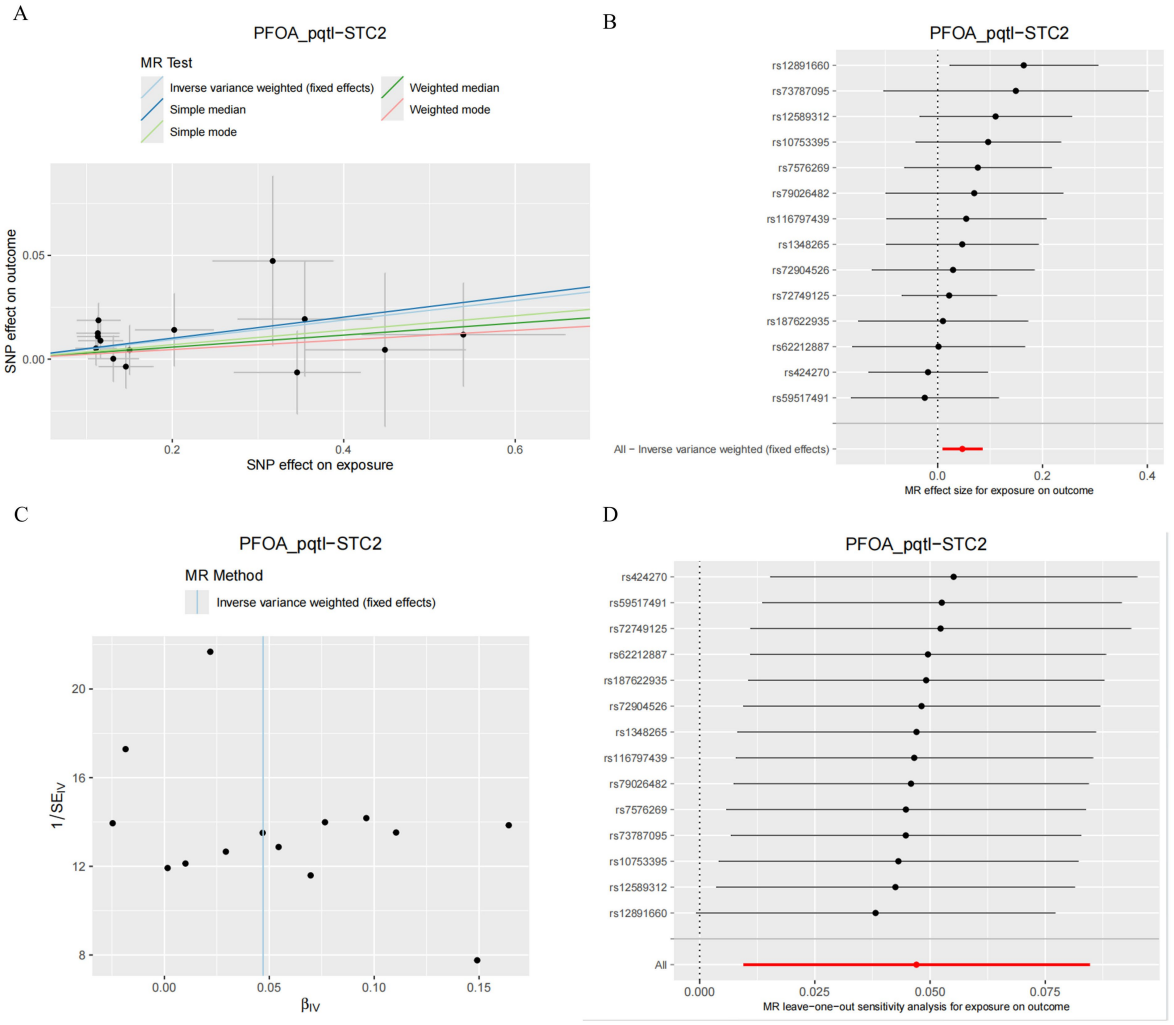
Sensitivity analysis of MR: PFOA–STC2–CRC. **(A)** Scatter plot of SNP effects showing alignment along the IVW slope, supporting a consistent causal estimate from PFOA to STC2. **(B)** Forest plot displaying individual SNP effect estimates and CIs, showing no single SNP strongly influences the MR result. **(C)** Funnel plot indicating symmetrical distribution of SNPs, suggesting no directional pleiotropy. **(D)** Leave-one-out analysis confirming the robustness of the causal effect; removal of any single SNP does not substantially alter the result.

Despite Sensitivity analyses, the PFOA-STC2 pairs proved to be a reliable method for verifying MR results, and MR Egger intercept tests demonstrated no horizontal pleiotropy (*p* = 0.173; Table 2). The absence of heterogeneity was proven by Cochran’s Q statistic (QP = 0.853; Table 3). The non-signaling nature of MR-PRESSO global tests (*p* = 0.87; Table 4.) was found to be absent. Unsolicited SNPs were identified in the one-missing-SNP analysis as driving the overall effect (Figure 10D). Gradient differences between exposure and outcome were confirmed through Steiger filtering, indicating that the causal pathway from exposure to outcome was correct (*p* < 1 × 10^−45^; Table 5). Test plots and scatterplots aided in verifying the correctness of the MR estimations (Figures 10A–C).

**Table 2:** Horizontal pleiotropy test (MR-Egger intercept).

Outcome	Exposure	Egger intercept	SE	*P*	Type
*STC2*	PFOA	0.0095	0.0066	0.173	pQTL

**Table 3:** Heterogeneity test for the PFOA–*STC2* association.

Outcome	Exposure	Method	Q	Q_df	Q_*P*
*STC2*	PFOA	MR-Egger	5.76	12	0.927
*STC2*	PFOA	IVW	7.86	13	0.853

**Table 4:** MR-PRESSO global test for pleiotropy.

Outcome	Exposure	MR PRESSO *P*	Type
*STC2*	PFOA	0.87	pQTL

**Table 5:** Steiger directionality test.

Exposure	Outcome	*R*^2^ (Exposure)	*R*^2^ (Outcome)	Correct direction	Steiger *p*-value
PFOA	*STC2*	0.0620	0.00044	TRUE	2.18 × 10^-50^

Measuring the mediation of STC2 in the causal relationship between PFOA and CRC revealed a total effect estimate of 0.116, with 0.010 mediated through STC2 representing 8.6% of the total effect (Table 6). Through a partially mediated pathway, STC2 expression may be upregulated by environmental exposure to PFOA, which may have an impact on CRC risk, according to these data.

**Table 6:** Mediation analysis results for PFOA → *STC2* → CRC.

Exposure	Mediator	Outcome	Total effect	Effect on mediator	Mediator effect on outcome
PFOA	*STC2*	CRC	0.116	0.047	0.213

### 3.13 RT-qPCR method to verify the mRNA expression of PRAME, CDKN2A, and STC2 in two colorectal cancer cell lines

**Figure 11:**
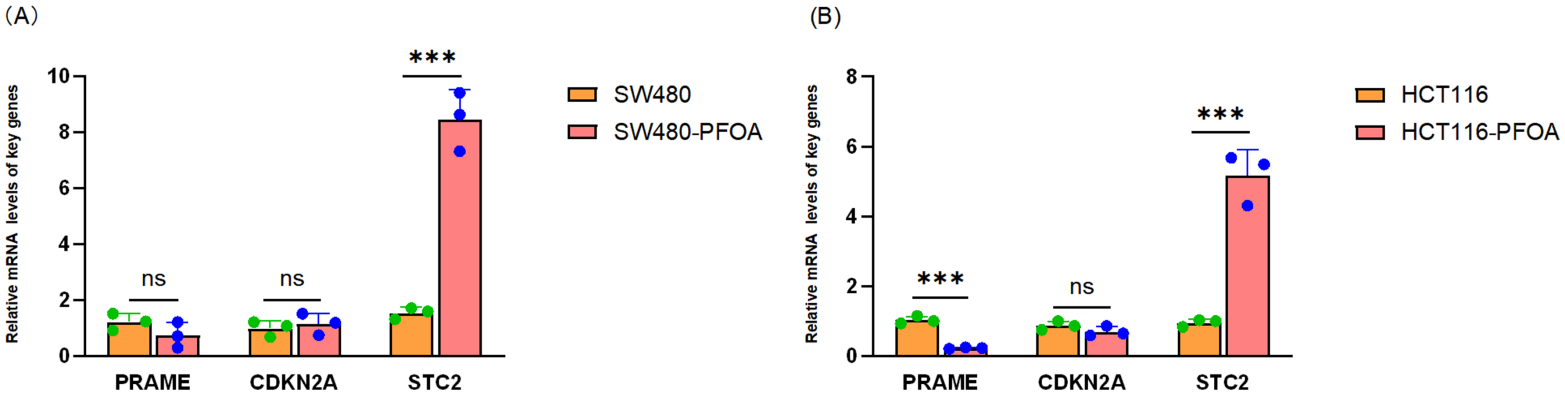
RT-qPCR method to verify the mRNA expression of PRAME, CDKN2A, and STC2 in two colorectal cancer cell lines. **(A,B)** PFOA promoted STC2 expression in SW480-PFOA and HCT116-PFOA. The relative mRNA PRAME, CDKN2A, and STC2 (*n* = 3 for control group and *n* = 3 for PFOA treatment group) levels in SW480 (human colorectal adenocarcinoma cells SW480) and HCT116 (human colorectal carcinoma cells HCT116) measured by RT-qPCR and shown as the mean ± SEM. Statistical significance at the level of *p < 0.05; **p < 0.01, ***p < 0.001, ****p < 0.0001 by two-independent-samples two-tailed *t*-test (*p* = 0.2051, *p* = 0.5908, *p* = 0.0004 for **A**, *p* = 0.0002, *p* = 0.1946, *p* = 0.0006 for **B**).

RT-qPCR was employed to determine the transcriptional levels of key genes PRAME, CDKN2A, and STC2 in two tumor models, SW480 and HCT116, following PFOA exposure. Data revealed significantly elevated STC2 mRNA expression in both PFOA-exposed cell lines, while CDKN2A transcription showed no statistically significant alteration (*p* > 0.05). However, PRAME transcription abundance failed to reach statistical significance in either the SW480-PFOA group (p > 0.05) or the HCT116-PFOA group (*p* < 0.001) (Figures 11A,B). These results indicate that among the three genes, only STC2 exhibits a consistent transcriptional response to PFOA exposure. This result suggests that the oncogene STC2 exhibits a broadly upregulated pattern in PFOA-treated CRC cell lines. These RT-qPCR findings showed high concordance with MR analysis, suggesting that STC2 may act as a key mediator linking PFOA exposure to CRC. Taken together, the evidence supports a potential role of STC2 in mediating PFOA-associated CRC progression, rather than implying that all identified genes are directly induced by PFOA.

## 4 Discussion

PFOA, a member of the perfluoroalkyl substances (PFASs), has been widely used in industrial production for decades. Although its use is now strictly regulated, its high chemical stability and environmental persistence allow it to remain in both natural ecosystems and human tissues (42). Amid growing concerns over the environmental pollution and health hazards posed by PFASs, many countries are taking steps to restrict their usage and are actively exploring safer alternatives. However, many novel PFAS variants retain the potential to adversely affect ecosystem integrity and human health (43). PFAS can be encountered by humans through dual routes: occupational activities and environmental dissemination. PFOA has been widely proven to affect the health of various organisms, including development, metabolism, and neurological function (44). Quantitative pooling of epidemiological studies demonstrates statistically significant connections between PFAS exposure and heightened susceptibility to several malignancies (45). The gastrointestinal tract constitutes the main barrier for the entry of these compounds through dietary intake and water consumption. Reduced fecal excretion efficiency of PFOA may contribute to its bioaccumulation in bodily organs such as the intestines (46). Previous *in vivo* studies have confirmed that PFOA and other PFASs compounds accumulate more significantly in intestinal tissues than in other organs, with PFOA concentrations in both the small intestinal and colonic regions showing a significant dose-dependent increase (47). The concentration of PFOA in small intestine and colon tissues showed a significant dose-dependent increase. PFOS disrupts the intestinal microbiota in mice, resulting in intestinal tissue damage, PFOA exposure may impair epigenetic gene expression and compromise intestinal barrier function (8). Therefore, exposure to perfluorinated compounds may be associated with colorectal cancer, but the exact mechanism between the two is unknown and remains an unexplored frontier in scientific research.

Univariate Cox regression and LASSO analysis were applied to the training dataset using the intersecting gene set, leading to the identification of three key prognostic genes: *PRAME*, *CDKN2A*, and *STC2*. A prognostic model was then constructed based on the expression levels of these genes. Risk scores were calculated for all patients in the training cohort and used to stratify them into high-risk and low-risk groups. Each gene demonstrated prognostic relevance for OS, and the model was validated across independent datasets. Correlational assessment of the established risk model and clinical characteristics revealed statistically significant distinctions in clinical manifestations when the risk-based classifications were compared (48). Univariate and multivariate Cox regression analyses showed that risk scores were independently associated with prognosis, and the predictive results of the gene prognostic line plots were consistent with the actual outcomes to a certain degree (49).

Data analysis identified *PRAME*, *CDKN2A*, and *STC2* as key gene targets associated with PFC exposure in CRC. However, experiments and MR analysis demonstrated that only STC2 is participated in the carcinogenic pathway of PFCs in CRC. Notably, although PRAME, CDKN2A, and STC2 were identified as prognostic genes through transcriptomic and survival analyses, only STC2 demonstrated a consistent response to PFOA exposure *in vitro*. This discrepancy highlights the important distinction between prognostic relevance and environmental responsiveness. The CTD-based screening and differential expression analysis primarily capture association-level relationships, which may include indirect or context-dependent genes rather than direct exposure-responsive targets. Therefore, the constructed model should be interpreted as a CRC prognostic model based on PFC-associated genes, within which STC2 represents the key mediator linking environmental exposure to tumor progression. In this study, in vitro validation experiments were conducted using PFOA as a representative perfluorinated compound. PFOA was selected because it demonstrated the most consistent and robust association with CRC risk in the Mendelian randomization analysis, and it has been more extensively characterized in previous experimental studies compared to other PFCs. Although PFOS was also identified as a relevant exposure factor in the MR analysis, it was not included in the in vitro experiments due to experimental constraints. Given the structural similarity and shared biological properties among PFCs, PFOS may exert comparable regulatory effects on STC2 expression, which warrants further investigation in future studies. In addition, the present study primarily focused on transcriptional regulation of STC2, and protein-level validation was not performed. Future studies incorporating protein expression analyses (such as Western blot or ELISA) will be necessary to further elucidate the regulatory effects of PFOA on STC2 at the translational level.

STC2 is a highly conserved glycoprotein that plays a key role in tumor progression and immune regulation. This protein is highly expressed in various cancers, including breast, liver, and colorectal cancers, and its expression is closely associated with tumor proliferation, invasion, migration, and resistance to radiotherapy and chemotherapy (50). As a downstream gene of hypoxia-inducible factor, STC2 is activated under hypoxic conditions and promotes tumor cell proliferation by modulating cyclin activity and promoting cell proliferation (51). The study revealed that STC2 is significantly upregulated in CRC tissues, and can promote CRC progression by regulating cancer cell invasion and proliferation (52). As a critical epitranscriptomic mechanism, m^6^A methylation actively participates in the initiation and evolution of neoplastic diseases (53). Existing literature supports that m^6^A regulatory factors, including YTHDC1 and WTAP, can suppress the proliferation, migration, and invasion of bladder cancer cells by modulating m^6^A levels (15). Furthermore, it has been demonstrated that PFOA exposure enhances the stability of MAPK15 mRNA by increasing m^6^A methylation, thereby activating lysosome-mediated autophagy and exacerbating the tumorogenic phenotype of prostate cancer cell (54). Methyltransferase-like 3 (METTL3), an RNA methyltransferase, positively regulates the m6A modification of STC2. In other words, the METTL3/STC2 axis could serve as a promising therapeutic target to combat resistance and metastasis in colorectal cancer (55). This suggests that PFCs may promote CRC progression by enhancing m6A-mediated upregulation of STC2, thereby contributing to both tumor aggressiveness and poor treatment response.

By intersecting these DEGs with the identified targets, we ultimately identified 331 genes that showed significant interactions. From the established PPI network, 20 key hub genes were identified, and created a Sankey diagram for intuitive visualization, KEGG pathway analysis revealed 134 significantly enriched pathways, particularly the JAK–STAT, PI3K-Akt, NF-κB, FoxO, and TNF signaling pathways, which are associated with CRC development and immune regulation. These pathways associate with malignant progression, immune regulation, and cellular stress responses. Among these, the PI3K/AKT signaling pathway has received particular attention. PFOA exposure demonstrates significant efficacy in enhancing neoplastic cell growth, with significant effects observed in both animal models and cell-based assays (56–58). Molecular interaction researches indicate that PFOA engages with PI3K via molecular interactions involving hydrogen bonding and electrostatic forces, thereby selectively activating the PI3K/AKT pathway. Once PI3K is activated, it initiates phosphorylation of AKT, which subsequently triggers a cascade of downstream signaling events involved in cell survival, growth, and proliferation, all of which are key processes in cancer development (59). The STC2 protein modulates the phosphorylation status of ERK1/2 kinases and VEGF factor, and activate PI3K, Initiate the cascade reaction of AKT downstream, These substances are believed to play a role in regulating the PLVAP protein of tumor necrosis factor (60, 61). Further research findings indicate that the high-risk score group constructed from the STC2 gene demonstrates significantly enhanced sensitivity to targeted drugs acting on the PI3K/AKT signaling pathway (62). Based on these findings, we propose that PFOA may promote CRC progression by regulating PLVAP expression via the STC2-mediated activation of the PI3K/AKT signaling pathway. Therefore, the STC2 target and PI3K/AKT signaling pathway can serve as therapeutic targets for intervening in CRC progression when PFCs are exposed.

Through GSEA analysis, multiple KEGG pathways were identified, including the Glutathione Metabolism, Chemokine Signaling Pathway, TGF- *β* Signaling Pathway, Nod-Like Receptor Signaling Pathway, Toll−Like Receptor Signaling Pathway, Wnt Signaling Pathway. Among the various mechanisms promoting epithelial-mesenchymal transition (EMT), multiple studies have confirmed that TGF-*β* serves as a critical driver for metastatic progression in multiple cancer types, including breast, prostate, pancreatic, and colorectal cancers (63). SMAD proteins in the cytoplasm are key targets of the TGF-β signaling pathway. These proteins are categorized into three types: receptor-regulated SMAD2/3 proteins, the inhibitory protein SMAD7, and the general SMAD proteins (64). Studies have demonstrated that reduced SMAD7 protein levels significantly potentiate cellular migration and invasion capacities in neoplastic cells (65). TGF-β/SMADs signaling is linked to the orchestration of EMT processes (66). Recent research has demonstrated that PFNA can modulate this pathway by altering the phosphorylation status of SMAD2/3 and regulating their nuclear translocation. Specifically, PFNA treatment induces a dose-dependent decrease in SMAD7 expression with synchronous upregulation of p-SMAD2/3. At environmentally realistic levels, PFNA triggers SMAD7 degradation through the ubiquitin-proteasome system, leading to subsequent TGF-β/SMAD pathway activation and promoting EMT in ovarian cancer (67). Research has demonstrated that GDF15, a member of the TGF-β superfamily, can activate the classical TGF-β signaling pathway (68), which subsequently stimulates and promotes endometrial mesenchymal transition (EndMT) (69). Notably, recent single-cell transcriptomic studies have revealed that the STC2 + malignant cell population is enriched for EMT-related gene signatures and plays a crucial role in tumor progression. These studies also found that TGFBR2, the receptor for TGF-β, is highly expressed in endothelial cells (70). STC2 is primarily found in endothelial cells (62). Therefore, we suggest that malignant cells expressing STC2 + may enhance the EMT program by activating this signaling pathway, thereby creating a favorable environment for tumor growth and exacerbating colorectal cancer.

Furthermore, immune microenvironment analysis intergroup disparities between the prognostic strata revealed differential abundance in 19 immune cell lineages, including CD4 + T helper cells, M2 macrophages, memory B cells, plasma cells, Tregs, B cells, CD4 + memory T cells, conventional dendritic cells (cDCs), and Th1 cells. In the TCGA-CRC dataset, further analysis showed that the expression of CDKN2A demonstrated strong positive correlations with M2 macrophage and Th1 cell populations, microvascular endothelial cells, and lymphatic endothelial cells, while showing marked negative correlations with plasma cells, T follicular helper cells, CD4 + memory T cells, and B cells. In contrast, STC2 expression exhibited a strong negative correlation with class-switched memory B cells, suggesting immunosuppressive or immune-evading properties. These findings suggest that PRAME, CDKN2A, and STC2 may participate in CRC progression by modulating immune cell infiltration, indicating their potential roles in tumor immune regulation (71). As pivotal modulators of immune responses, checkpoints employ regulatory factor to preserve immunological equilibrium, safeguarding host tissues against immunopathological injury. In cancer treatment, immune checkpoint inhibitors have become a pivotal immunotherapy strategy by neutralizing tumor cell-mediated immune suppression and reactivating T cells to attack malignant tumors. Based on the training set TCGA-CRC, which contains CRC patient samples with survival information, checkpoints such as *ASXL1*, *CD244*, *CDK4*, *DOT1L*, *FAS*, *PLK1*, *MCL1* and *MDM2* are significantly correlated with risk scores, suggesting that it may be related to immune checkpoints (33). Risk models have practical significance in clinical prognosis assessment, including helping high-risk patients to screen, guide immunotherapy and select targeted drugs.

Our findings demonstrate a potential link between environmental PFC exposure and colorectal cancer risk through STC2-mediated mechanisms. However, the generalizability of these results should be interpreted with caution. The transcriptomic and clinical data were primarily derived from the TCGA cohort, which consists predominantly of patients of European ancestry, while the MR analysis was also based on European populations (FinnGen). Therefore, population-specific genetic backgrounds, environmental exposure patterns, and lifestyle factors may influence the applicability of our findings to other populations. Future studies in more diverse cohorts are needed to validate the robustness and cross-population generalizability of these results.

From a public health perspective, our study provides novel evidence supporting the potential carcinogenic role of perfluorinated compounds in colorectal cancer. Given the widespread environmental persistence and bioaccumulation of PFCs, these findings highlight the importance of environmental exposure assessment in cancer prevention strategies. In particular, STC2 may serve as a potential biomarker linking environmental exposure to tumor development, offering opportunities for early risk stratification and targeted intervention. Moreover, our findings underscore the need for stricter regulation and monitoring of PFCs in the environment, as well as increased awareness of their potential long-term health effects. Integrating environmental exposure data with molecular and clinical information may further advance precision medicine approaches in colorectal cancer.

This study systematically investigated potential PFC targets and mechanisms in CRC using bioinformatics approaches. However, several limitations should be acknowledged. First, variability in data sources, algorithms, and screening criteria may lead to heterogeneity in the quality of PFC-target associations, resulting in a lack of standardization across studies in this field. Second, the binding affinities between PFCs and their predicted target proteins were primarily inferred from computational simulations, and lack sufficient experimental validation, including clinical trials. Additionally, while some of the identified targets and pathways are promising, the causal relationships remain unclear due to insufficient functional evidence. Furthermore, the QTL datasets used in this study were primarily derived from blood or plasma rather than colon tissue, which may not fully capture tissue-specific regulatory mechanisms in colorectal cancer. It should be noted that the concentration of PFOA used in this study (10 μM) is higher than typical environmental exposure levels in humans. However, such concentrations are commonly employed in *in vitro* studies to mimic high-dose or occupational exposure scenarios and to ensure detectable biological responses. Therefore, the findings should be interpreted with caution when extrapolating to real-world exposure conditions. In addition, due to the limited availability of detailed clinical variables (such as tumor differentiation grade, MSI status, and treatment information) in the TCGA dataset, residual confounding factors cannot be fully excluded in the prognostic analysis. The future research direction of environmental toxins and CRC should reveal the mechanism of PFC and gene regulation network, expand the precision oncology research under environmental exposure background, promote the transformation of risk model to clinical by combining multi-omics and clinical data.

This study identified three prognostic genes related to PFC in CRC — PRAME, CDKN2A, and STC2—through the analysis of general transcriptome data, and developed a predictive model for prognosis assessment. The generated risk stratification system exhibits strong predictive power and independent prognostic value in the training set. Based on functional enrichment and immunological analysis, STC2-related signaling pathways play a significant role in tumourigenesis, disease progression, and immune evasion mechanisms. This risk model can screen high-risk patients, guide immunotherapy, and select targeted drugs by leveraging immune checkpoints such as ASXL1, CD244, and CDK4. Building on this, we further validated through Mendelian randomization analysis and RT-qPCR that STC2, a colorectal cancer-associated PFC regulator, is markedly elevated in CRC cells and contributes significantly to PFC-associated colorectal tumorigenesis.

## Data Availability

The datasets presented in this study can be found in online repositories. The names of the repository/repositories and accession number(s) can be found in the article/supplementary material.

## Footnotes

1
http://ctdbase.org/
